# Spatio‐Temporal Modeling of Land‐Use/Land‐Cover Change and Land Surface Temperature Using SVM and CA–Markov in Dilla Town, Ethiopia

**DOI:** 10.1002/gch2.70103

**Published:** 2026-04-10

**Authors:** Tsion Ayalew Kebede, Wondimu Haimanote Gebremariam, Hiwot Yitayew Bogale, Talema Moged Reda, Muralitharan Jothimani

**Affiliations:** ^1^ Department of Land Administration and Surveying Dilla University Dilla Ethiopia; ^2^ Department of Remote Sensing Space Science and Geospatial Institute Addis Ababa Ethiopia; ^3^ Department of Geology College of Natural and Computational Sciences Arba Minch University Arba Minch Ethiopia

**Keywords:** change detection, Dilla, Ethiopia, machine learning classification, prediction modeling, thermal remote sensing, urban expansion, urban heat island

## Abstract

Urban expansion in Dilla Town, Ethiopia, has progressively reshaped local thermal conditions by replacing vegetation and cropland with impervious built‐up surfaces. This study analyses how three decades of land‐use/land‐cover (LULC) change have modified land surface temperature (LST) and projects future thermal patterns. Landsat TM/ETM+/OLI imagery from 2001, 2011, and 2021 was classified into five LULC classes using a Support Vector Machine (overall accuracy 90.4–92.4%; κ = 0.88–0.90). LST was retrieved from thermal bands via a single‐channel algorithm and related to NDVI and the Normalized Difference Built‐up Index (NDBI). Between 2001 and 2021, the built‐up area increased by ∼5.34 × 10^6^ m^2^, while mixed forest and cropland decreased by 3.14 × 10^6^ m^2^ and 2.92 × 10^6^ m^2^, respectively. Over the same period, LST rose from 23.17°C to 33.80°C to 24.42°C to 40.18°C, and mean LST increased from 27.47°C to 30.61°C. In 2021, LST showed a strong positive correlation with NDBI (R^2^ ≈ 0.64) and a negative correlation with NDVI (R^2^ ≈ 0.36). A CA–Markov model, validated with a Kappa Index of Agreement of up to 0.95, predicts continued urban expansion and a marked shift toward hotter LST classes by 2031, emphasizing the need for greening, wetland conservation, and heat‐aware urban planning.

## Graphical abstract

Multi‐temporal Landsat imagery (2001, 2011, 2021) was used to map land use/land cover and retrieve land surface temperature, quantify change, and model future scenarios. Results indicate rapid expansion of built‐up land accompanied by declining mixed forest and cropland and increasing thermal hotspots. CA–Markov projections suggest continued urban growth and higher temperatures by 2031, supporting targeted urban planning.

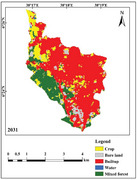

## 1 Introduction

Land use and land cover (LULC) changes the properties of the ground surface and the exchange of energy, water, and materials, which affects climate at local to global scales and disrupts biogeochemical processes [1, 2, 3, 4, 5]. The climatic effects vary from one region to another, depending on the interaction between the land and the atmosphere, which is affected by local climate, environmental conditions, terrain, and vegetation [6, 7, 8]. This variability drives widespread deforestation, accelerates soil erosion, degrades land quality, and ultimately reduces biodiversity [9]. LULC change primarily affects local‐to‐regional surface energy balance (albedo, evapotranspiration, and heat storage), which can intensify local warming/urban heat island effects [10, 11]. In Ethiopia, LULC change is high and increasing [12, 13], due to population increase and land management perceptions [14, 15], with further exposure to runoff, flooding, and sedimentation [16, 17]. Urban LULC change increases LST and gives rise to urban heat islands [8, 18, 19, 20, 21, 22]. Over the last decades, seasonal average temperatures in Ethiopia have risen [16], while Belg and Kiremt rainfall in parts of southern and southeastern Ethiopia have declined [23], increasing urban energy demand and pollution‐related health risks [24].

Temperature variability is a burden for urban systems—increasing energy demand and health risks [24, 25, 26]. Converting vegetation to impervious surfaces increases LST by altering evapotranspiration and the partitioning of surface water and energy, with additional adverse effects [27, 28, 29, 30, 31]. Machine‐learning methods—Support Vector Machine (SVM), Artificial Neural Network (ANN), Random Forest (RF), and Logistic Regression (LR)—are widely used [32, 33], many of which are nonparametric, fast to apply, provide interpretable rules, and support continuous/nominal data [34, 35]. SVMs are often better than alternatives and are good with small training sets and fine‐scale compositional classes [28, 36]; in the case of dynamic rural‐urban mosaics, they are particularly effective [37]. Accordingly, SVM was used in this study to classify five LULC types (2001, 2011, 2021).

Satellite RS provides near‐real‐time, spatially extensive LST products [36, 38] and is effective at quantifying the impacts of land cover and urbanization [39, 40]. (NDBI), The Normalized Difference Bareness Index (NDBaI) and the Normalized Difference Water Index (NDWI)—both based on optical bands—are commonly used in urban heat island (UHI) studies. Because land surface temperature (LST) is highly correlated with LULC metrics, variability in NDVI and NDBI can be used to predict surface temperature [41]. NDBI uses spectral contrasts in the urban fabric to define built‐up areas [42, 43]; NDBaI uses shortwave infrared (SWIR) and thermal infrared (TIR) to highlight bare surfaces. NDVI—computed from red and near‐infrared (NIR) bands—captures vegetation fraction; more vegetation usually moderates temperature variability [44, 45, 46].

Modeling LULC‐land surface temperature (LST) dynamics ranges from multi‐agent, hybrid, analytical, expert, statistical, evolutionary, cellular, and Markov approaches [47, 48, 49, 50]. Markov chains are ideal for predicting changes in environmental states and inferring thermal responses to vegetation changes [50]. Empirically, CA‐Markov with Landsat and supervised classification has been used to predict seasonal LST [51], and combined with ANN to estimate the landscape risk [52]; CART with a single‐channel algorithm has been used to quantify the effects of LUC on LST and predict trends [53, 54]. RS and GIS integration is established for tracking urban expansion and its thermal effects; broad spatial LST coverage allows evaluation of anthropogenic surface modifications, and GIS classification identifies the type, rate, and distribution of growth and associated surface warming [55]. Sparse ground stations can reduce the spatial representativeness of the LST models, highlighting the need for RS.

The general objective of this study is to analyze and model the spatio‐temporal dynamics of LULC change and LST in Dilla Town, Ethiopia, using satellite remote sensing and geospatial modeling techniques. Specifically, the study aims to: (i) classify and map LULC for 2001, 2011, and 2021 from multi‐temporal Landsat imagery using a SVM classifier; (ii) retrieve and characterize the spatial and temporal variability of LST over the same period; (iii) quantify the relationships between LST and key land‐surface indicators such as NDVI, NDBI and related indices, as well as across different LULC classes; and (iv) simulate future LULC patterns and the associated LST distribution using a CA–Markov framework to generate forecasts for the coming decades.

This study is needed because rapidly expanding secondary cities such as Dilla are experiencing intense land conversion from vegetation and agricultural land to built‐up surfaces, with limited empirical evidence on how these changes are altering the local thermal environment, energy demand, and climate‐related health risks. Existing research on LULC–LST interactions in Ethiopia has concentrated mainly on major metropolitan areas [37], leaving a critical knowledge gap for intermediate urban centers that are growing fastest yet have the weakest planning and monitoring capacity. The novelty of the study lies in its integrated use of an SVM classifier with CA–Markov modeling to jointly reconstruct past LULC trajectories and forecast future land‐cover states, and then propagate these changes to predict future LST patterns via satellite‐derived indices. By coupling empirically calibrated LST–index relationships with scenario‐based LULC simulations over a 20‐year historical period, the study provides a process‐oriented and transferable framework for anticipating emerging urban heat hotspots in data‐scarce African cities.

## 2 Materials and Methods

### 2.1 Study Area

**FIGURE 1:**
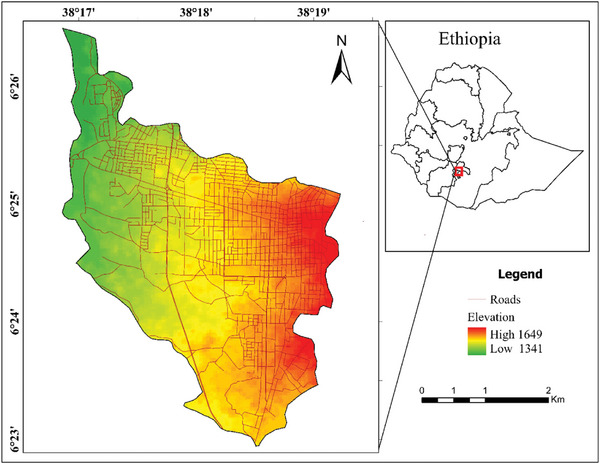
The study area map.

The investigation was conducted in Dilla town (Figure 1), the administrative capital of Gedio Zone in southern Ethiopia. Dilla lies 365 kilometers from Addis Ababa, the capital of the country, at latitudes 6° 22' to 6° 42' N and longitudes 38° 21' to 38° 41' E. The height of the town ranges from 1341 to 1649 m above mean sea level (Figure 1). The average annual precipitation varies between 250 and 1400 mm. At the same time, the average annual temperature ranges from 23°C to 30°C. The study region covers approximately 1210.89 km^2^ and has a population of 97 516 individuals.

### 2.2 Data Sources and Pre‐Processing

Landsat imagery (30 × 30 m) was downloaded for the study years 2001, 2011, and 2021, following multi‐temporal remote sensing strategies commonly adopted in advanced segmentation and land‐cover mapping studies [56] from the US Geological Survey (USGS) and National Aeronautics and Space Administration (NASA) EarthExplorer (https://earthexplorer.usgs.gov). All the selected scenes had less than 10% cloud cover. Landsat was chosen as it is widely available and used in LULC and LST analysis. Layer stacking, image augmentation, band combinations, and false‐color composite production were all part of the preprocessing steps. All images were projected to WGS 1984 / UTM Zone 37N to maintain spatial consistency. Thermal bands (TM Band 6 and OLI/TIRS Band 10) were employed to derive at‐sensor brightness temperature. The LULC in the study area was categorized into five classes: mixed forest, cropland, water bodies, urban built‐up areas, and barren land (Table 1). Landsat 5 TM and Landsat 8 OLI/TIRS datasets were used for both LULC classification and LST extraction (Table 2). Ground control points collected using handheld GPS units were applied to assess classification accuracy. In addition, LST, NDBI, and NDVI were computed annually to examine how LST varies with LULC dynamics.

**TABLE 1:** Description of LULC classes.

LULC class	Description/Definition	Typical characteristics	Examples
Mixed forest	Areas dominated by mixed tree species with dense canopy cover.	High NDVI; strong NIR reflectance; generally lower LST than built‐up and barren surfaces.	Forest patches; riparian woodland; dense tree cover.
Cropland	Agricultural land used for seasonal or perennial crops; includes cultivated and fallow fields.	Moderate‐high NDVI depending on season; patchy texture; variable reflectance by crop stage.	Cultivated fields; smallholder farms; fallow plots.
Water bodies	Permanent or seasonal open water (rivers, ponds, lakes, reservoirs).	Very low NIR/SWIR reflectance; high NDWI; typically lowest LST.	Rivers; ponds; reservoirs; open‐water wetlands.
Urban built‐up areas	Impervious surfaces such as buildings, roads, and paved infrastructure.	Higher visible/SWIR reflectance; elevated NDBI; sharp edges/linear patterns; typically higher LST.	Residential/commercial areas; roads; industrial zones.
Barren land	Exposed soil, sand, rock, or degraded sparsely vegetated surfaces.	Low NDVI; high SWIR reflectance; high bareness indicators (e.g., NDBaI); often higher LST.	Bare soil; rocky ground; construction/exposed land.

**TABLE 2:** Data and their sources.

No	Satellite/Sensor	Pixel size	Spectral resolution	Band used	Date
1	Landsat 5	30 m	Multispectral (8 bands)	1,2,3,4,5,7	January 2001
2	Landsat 5	30 m	Multispectral (8 bands)	1,2,3,4,5,7	January 2011
3	Landsat 8	30 m	Multispectral (8 bands)	1,2,3,4,5,6,7,9	January 2021

## 3 Methods

### 3.1 LULC Classification and Its Accuracy Assessment

Supervised LULC mapping was performed using Support Vector Machine (SVM) for preprocessed Landsat images for 2001, 2011, and 2021. The SVM workflow consisted of three steps: (i) manual digitization of polygons that outline the training space of each class; (ii) Regions of Interest (ROIs) delineation and recording of training areas as ROIs in a spatial framework solely based on pixel values; and (iii) pixel‐wise assignment of class labels using the ROIs. The SVM, which is nonparametric and does not require normally distributed inputs, found an optimal separating hyperplane after selecting a kernel‐specific error penalty parameter; per‐pixel spectral signatures were compared with the training samples to produce the classified maps. Five LULC categories were considered: urban/built‐up areas, vegetation, forest, barren land, and water bodies. To achieve adequate class separability, 250 training samples per image were delineated using the ROI tool in ENVI.

Class definitions followed those used in this study: urban/built‐up areas include residential, industrial and commercial buildings, mixed‐use structures, roads, and transport infrastructure; vegetation comprises agricultural and horticultural fields, scrublands, croplands, and fallow land; forest consists of coniferous stands and other tree plantations; barren land denotes exposed or sparsely vegetated surfaces, including areas such as landfills; and water bodies include open water, rivers, and streams.

Classification performance was evaluated using standard accuracy statistics that quantify the correctness of pixel‐level LULC assignments. At least 50 reference samples per class were used, yielding a total of 250 validation points (50 for each class). Reference data were derived from visual image interpretation, GPS‐based field surveys, and high‐resolution Google Earth imagery. From the resulting confusion matrix, omission error (OE), commission error (CE), overall accuracy (OA), and the Kappa coefficient (K) were computed. Producer's accuracy represents the proportion of reference pixels correctly classified for a given class, whereas user's accuracy reflects the reliability of the mapped class on the ground. OA is defined as the ratio of correctly classified pixels (sum of the main diagonal) to the total number of pixels, and K quantifies the level of agreement between the classification and the reference data beyond that expected by random chance.

### 3.2 Land‐Use and Land‐Cover Change Detection

LULC change was quantified using a post‐classification, matrix‐based approach. Classified maps were produced in ENVI using predefined settings, and change information was extracted using transition matrices spanning the full analysis period (2001–2021). Transition matrices were generated in ArcGIS for two decadal intervals (2001–2011 and 2011–2021) to assess relative changes among LULC categories. Each transition matrix is a square table with all LULC classes represented in both the rows (initial state) and the columns (subsequent state); summaries are reported in square meters and percentages. The formula used to determine percentage changes in LULC is given in Equation (1).

(1) $$\mathrm{LULC}\;\mathrm{change}\;\left(\%\right)=\frac{\left(A_{2}-A_{1}\right)}{\left(A_{1}\right)}\;*100$$

“where *A_1_* is the area of each LULC class for the initial image, *A_2_* is the area of each LULC class for the later image. The LULC change rate was computed using Equation (2).”

(2)

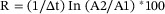

“where R is the annual rate of change in %, *∆*t is the time interval in years during the LULC change being assessed, ln is the base of the natural logarithm function, *A_1_* is the area of each LULC class for the initial image, and *A_2 is the_* area of each LULC class for the later image.”

### 3.3 Ndvi

NDVI was employed to characterize green vegetation cover [45, 46] and used as an explanatory variable in land surface temperature (LST) analysis [24, 57, 58]. NDVI is derived from the red (RED) and near‐infrared (NIR) spectral bands [59], with values from the Landsat imagery ranging between −1 and 1, and was computed using Equation (3):

(3) $$\mathrm{NDVI}\;=\frac{\mathrm{NIR}-\mathrm{RED}}{\mathrm{NIR}\;+\;\mathrm{RED}}$$

“where: NIR is the near‐infrared band (TM band 4, OLI band 5) as well as the red band (TM band 3, OLI band 4).”

### 3.4 Ndbi

The NDBI enhances the identification of built‐up land in remote sensing imagery. When used with an appropriate threshold, it enables adequate delineation of urbanized areas, which are typically associated with higher LST in metropolitan environments. NDBI is computed from the normalized difference between the short‐wave infrared (SWIR1) and near‐infrared (NIR) bands, thereby emphasizing impervious and developed surfaces, as shown in Equation (4).

(4) $$\mathrm{NDBI}\;=\frac{B_{\mathrm{SWIR}1}-B_{\mathrm{NIR}}}{B_{\mathrm{SWIR}1}+B_{\mathrm{NIR}}}$$

“where SWIR‐Short Wave Infrared band and NIR‐Near‐Infrared Band.”

### 3.5 Land Surface Temperature Retrieval

LST was computed from Landsat thermal bands acquired in 2001, 2011, and 2021. Any object can emit thermal electromagnetic radiation, allowing data obtained by thermal sensors to be converted into sensor radiation. Equation (5) was used to convert values to spectral radiance.

(5) $$L\lambda =M_{L}Q_{\mathrm{cal}}+A_{L}-O_{i}$$

“where Lλ is the Top‐of‐Atmosphere (TOA) spectral radiance, ML is the band‐specific multivariate rescaling factor from the metadata, AL is the band‐specific additive rescaling factor from the metadata, Qcal is the calibrated standard product pixel value (DN), and Oi is the calibration offset for Landsat 5 band 6 and Landsat 8 TIRS band 10”.

Second step, the spectral radiance Lλ value was changed into brightness temperature TB by using Equation (6)

(6) $$T=\frac{K_{2}}{ln\;{(}_{L\lambda }^{K_{1}}+1)}$$

“where TB denotes the at‐satellite brightness temperature (K), Lλ the spectrum radiance, and K1 and K2 the two calibration constants K1 and K2 values are 607.76 and 1260.56 for Landsat 5 (TM), and 772.88 and 1321.07 for Landsat 8 (OLI), respectively. The Landsat 8 (OLI/TIRS) data contain two thermal bands (10 and 11), but only band 10 was used for LST estimation”.

Subsequently, to derive physically realistic land surface temperature from the blackbody assumption, the surface spectral emissivity (ε) (Equation 7) must be corrected. This is accomplished by applying pixel‐wise emissivity values estimated from NDVI for each image pixel.

(7) ε=0.004∗pv+0.986

where ε is the Land surface emissivity and P*v* is the proportion of vegetation, and was calculated by the formula in Equation (8)

(8) $$Pv={\left(\frac{NDVI-NDVI_{min}}{NDVI_{max}-NDVI_{min}}\right)}^{2}$$

The LST was calculated over the study area from Landsat images using Equation (9),

(9) $$LST=\left(\frac{TB}{1+\left(\lambda *\rho \right)*\ln\varepsilon }\right)$$

“where LST is Land Surface Temperature, TB is Brightness Temperature in Kelvin, λ is the wavelength of the emitted radiance (11.457 for TM and 11.269 for ETM+), ρ is 1.438^−2^ mK, and 𝜀 is land surface emissivity”.

Finally, the temperature values expressed in Kelvin were converted to degrees Celsius (°C) using Equation (10) (Cristóbal et al., 2018).

(10) $$T\left(C^{0}\right)=T\left(K\right)-273.15$$

### 3.6 Prediction of LST by Selecting Variables

Accurate estimation of LST requires robust relationships between surface temperature and predictor variables that are not affected by multicollinearity. Accordingly, land‐cover indicators were used as explanatory variables to evaluate their correlation with LST. A linear regression model was used, employing the indices with the most robust correlations. The interrelationships among the variables were examined to exclude predictors that exhibit strong clustering, thereby minimizing the risk of collinearity‐induced errors. The model's effectiveness was evaluated by applying it to predict the observed LST in 2021, and accuracy was measured using the Mean Absolute Percentage Error (MAPE) (Equation 11).

(11) $$MAPE\;\;\left(\%\right)=\frac{1}{N}\sum_{i=1}^{1}\left(\frac{T_{predicted_{i}-\;}T_{observed_{i}\;}}{T_{observed_{i}\;}}\right)*100$$

where T predicted and T observed are: LST computed from NDBI and retrieved from Landsat images. Following the accuracy assessment, the model was applied to estimate the spatial distribution of LST at 10‐year intervals. A decadal interval was selected because the analysis indicated pronounced fluctuations in LST across corresponding time periods.

### 3.7 Prediction of LULC Change and Validation of the Model

A wide range of predictive models has been developed to evaluate seasonal dynamics in LULC [33]. The Markov model, a stochastic framework, was employed to quantify the likelihood of transitions between land cover classes and thus characterize LULC dynamics over time [48, 60]. Stochastic variability is estimated by using time‐series methods to compute transition probabilities from historical observations over a specified period. The model permits the transition from state i to state j in a defined time interval T and was chosen for its suitability for forecasting land‐use and land‐cover changes, as well as the evolution of complex systems over time [53, 61]. The results of the Markov model are determined by its transition probabilities [48]. The transition probability (Pij) between states j and i represents the probability for land cover class i (in pixels) to change to land cover class j at time x + 1. This model uses Equations (12) and (5) to calculate the dynamics of change for each study region, using previous or existing land cover conditions [62, 63], as modified by [64, 65].

(12) $$L_{\left(X+1\right)}=P_{ij}\;*L_{\left(X\right)}$$

The transition probabilities are obtained from the transition samples collected over a specific time interval and are presented in the transition matrix in Equation (13).

(13) $$P_{ij}=\lfloor \begin{array}{cc} P_{11} & P_{12}\\ P_{21} & P_{22}\\ P_{m1} & P_{m2} \end{array}\;\begin{array}{cc} P_{13} & \text{…}.\;P_{1m}\\ P_{23} & \text{…}.\;P_{2m}\\ P_{m3} & \text{…}.\;P_{mm} \end{array}\rfloor \;$$

where L(x+1) and L(x) represent the land use/cover conditions at times (x+1) and (x), respectively. The transition probability matrix is (i, j = 1, 2, 3, m) with 0 _ Pij < 1 and Pm j = 1 Pij = 1, (i, j = 1, 2, 3, m). Markov chain analysis was applied to derive transition matrices and corresponding probabilities for LULC changes from 2001 to 2011 and from 2011 to 2021, which were then used to forecast future land‐use and land‐cover dynamics. However, the Markov chain model does not explicitly incorporate spatial information; it neither provides a mechanistic explanation of the processes driving change nor represents the geographical configuration of LULC classes, which is essential for reproducing realistic land‐cover patterns [48].

CA is widely used to forecast LULC dynamics because it can represent complex, spatially distributed systems. A CA model is characterized by five basic elements: the cell, the cell space, the neighborhood, time, and transition rules. Owing to their structural simplicity and internal consistency, CA models are especially effective in simulating the spatial distribution of future urban expansion and its impact on LST. In CA‐Markov implementations, Markov transition probability matrices are used in combination with current or past LULC states and the conditions of surrounding cells to derive spatially explicit transition rules and predict future LULC patterns [66]. This integration makes cellular automata a powerful and widely used approach to dynamic modeling in geographic information systems and remote sensing.

Nonetheless, standalone CA models are limited by their rule formulation and fixed model structure. Therefore, integrating complementary empirical and dynamic approaches, such as the CA–Markov model, is essential for robust dynamic spatial modeling of LULC [50, 60]. In this study, the LULC map simulated by the CA–Markov model was validated against the 2021 LULC map produced using SVM classification. The Kappa Agreement Index (KIA) was used to evaluate the CA–Markov model's performance in 2021 by comparing the supervised SVM‐derived LULC map with the corresponding simulated LULC map from 2021. The model was calibrated using LULC maps from 2001 and 2011 to reconstruct the 2021 LULC configuration and subsequently to project LULC changes for 2031. Following validation of the CA–Markov model, LULC patterns from 2001 to 2021 were used as inputs to forecast the LULC distribution for 2031.

### 3.8 Prediction of LST Using CA Markov Chain

The NDBI emerged as the most influential predictor of LST spatial patterns. It was computed from Landsat imagery for the years 2001, 2011, and 2021. NDBI maps for 2001 and 2021 were incorporated into the Markov chain model to generate transition probability (change likelihood) matrices, which were then used within the CA framework to simulate the index state for 2021. Subsequently, mean NDBI values derived from 2011 and 2021 were applied to the CA–Markov outputs to forecast NDBI conditions for 2031. The resulting NDBI maps were discretized into five distinct classes to represent different LST regimes. Finally, the projected NDBI values were converted to LST distributions using a linear regression model, as specified in Equation (14).

(14) LST=16.34∗NDBI+30.07

**FIGURE 2:**
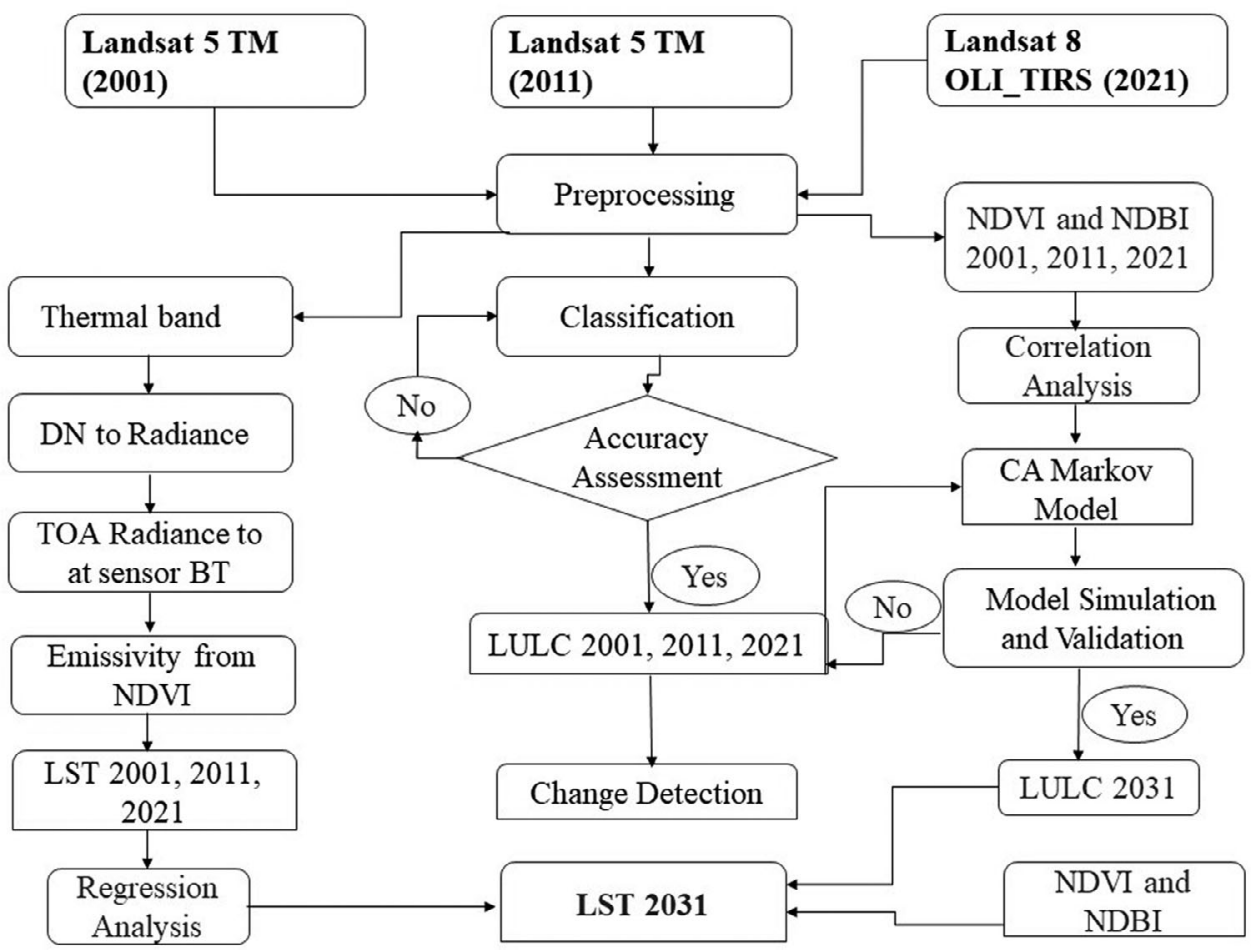
Methodology flow chart.

The collected LST maps were used to create (23°C–26°C, 26°C–30°C, 30°C–33°C, 33°C–36°C, and 36°C–41°C). These LST categories were explicitly chosen to demonstrate how LST distributions evolved from 2001 to 2021. To calculate the future LST of the research area for 2031, the estimated NDBI for that year were used. The overall methodology is shown in the flowchart (Figure 2).

## 4 Results and Discussion

### 4.1 Land Use Land Cover Classification

**FIGURE 3:**
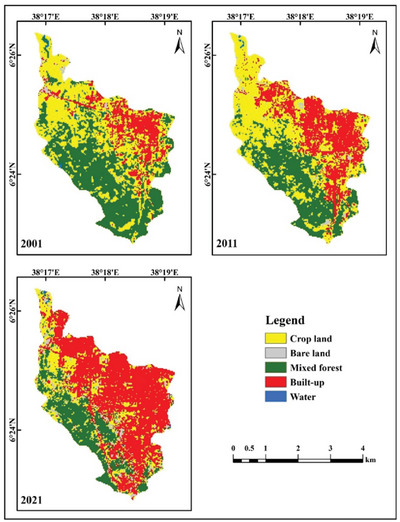
LULC classification maps for the years 2001, 2011, and 2021.

**FIGURE 4:**
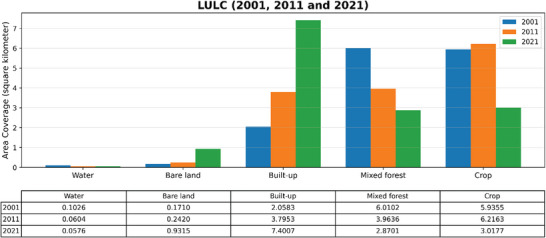
Area coverage of LULC for 2001, 2011, and 2021.

This study generated five land use and land cover categorization maps for 2001, 2011, and 2021, as illustrated in Figures 3 and 4, and analyzed spatiotemporal area coverage in Table 3 from 2001 to 2011. The extent of water bodies and mixed forests diminished by 0.0422 and 2.0466 km^2^, respectively. The coverage of urban areas, agricultural land, and unutilized land rose by 1.7370, 0.2808, and 0.0710 km^2^, respectively. From 2011 to 2021, the areas of water bodies, mixed forests, and farmland decreased by 0.0028, 1.0935, and 3.1986 km^2^, respectively. The expanse of constructed and unoccupied land increased by 3.6054 and 0.6895 km^2^, respectively (Table 3). Between 2001 and 2021, built‐up areas and vacant land increased by 5.3424 and 0.7605 km^2^, respectively, whereas water bodies, agricultural land, and mixed forest decreased by 0.0450, 3.1401, and 2.9178 km^2^, respectively.

**TABLE 3:** Area coverage of LULC between 2001 and 2021.

LULC class and area coverage (km^2^) (2001–2021)			
Land use types	2001	2011	2021
Water	0.1026	0.0604	0.0576
Bare land	0.1710	0.2420	0.9315
Built‐up	2.0583	3.7953	7.4007
Mixed forest	6.0102	3.9636	2.8701
Crop	5.9355	6.2163	3.0177
Total	14.2776	14.2776	14.2776

### 4.2 Accuracy Assessment for Classified LULC Map

Accuracy assessment was conducted to quantify classification errors and assess the reliability of the LULC maps. Figure 3 shows the classification outputs for five major land‐use/land‐cover classes for 2001, 2011, and 2021, based on 50 randomly selected reference points per class, for a total of 250 reference samples. Classification performance metrics were calculated with test data created by the trained SVM model. Independent samples, distinct from those used for training, were used for quantitative validation, allowing calculation of the overall accuracy and the Kappa coefficient (Table 4).

**TABLE 4:** Land use land cover change detection.

Land use land cover change in (km^2^) from 2001 to 2021						
Land use types	2001	2011	2021	Change from 2001 to 2011(km^2^)	Change from 2011 to 2021(km^2^)	Change from 2001 to 2021(km^2^)
Water	0.1026	0.0604	0.0576	−0.0422	−0.0028	−0.0450
Bare land	0.1710	0.2420	0.9315	0.0710	0.6895	0.7605
Built‐up	2.0583	3.7953	7.4007	1.7370	3.6054	5.3424
Mixed forest	6.0102	3.9636	2.8701	−2.0466	−1.0935	−3.1401
Crop	5.9355	6.2163	3.0177	0.2808	−3.1986	−2.9178
Total	14.2776	14.2776	14.2776			

The resulting LULC maps were compared with reference data and field‐based ground truth using an error (confusion) matrix to evaluate and summarize classification accuracy. Producer's accuracy was maximal for water bodies (100% in all three reference years), whereas the minimum producer's accuracy occurred for barren land (84.21%) in 2001. User's accuracy for barren land reached 98% in both 2013 and 2021, and was 80% for water bodies. Overall classification performance was satisfactory, with accuracies of 90.4%, 92.4%, and 92.0% for 2001, 2011, and 2021, respectively. The corresponding Kappa (K) statistics were 0.88, 0.90, and 0.90 for the study area in 2003, 2013, and 2021, respectively (Table 5), indicating strong agreement between the classified maps and the reference data.

**TABLE 5:** Accuracy assessment.

	Producer accuracy (%)					User accuracy (%)						
LULC class	1	2	3	4	5	1	2	3	4	5	Overall accuracy	Kappa (K)
2001	100	91.3	85.1	93.75	84.21	80	94	92	90	96	90.4	0.88
2011	100	95.45	88.9	95.74	84.48	94	84	96	90	98	92.4	0.9
2021	100	93.47	88.9	95.65	84.48	92	86	96	88	98	92	0.9

Where 1 = Water, 2 = Crop land, 3 = Mixed forest, 4 = Buit‐up, and 5 = Bare land

### 4.3 Normalized Difference Vegetation Index

**FIGURE 5:**
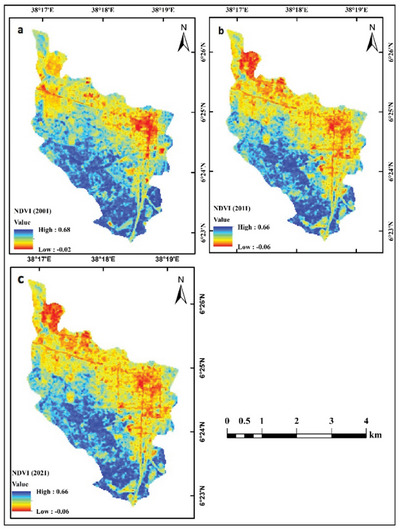
NDVI maps in (a) 2001, (b) 2011, and (c) 2021.

The NDVI analysis derived from Landsat imagery exploits the characteristic spectral response of vegetation, which strongly absorbs visible radiation for photosynthetic activity and reflects near‐infrared (NIR) radiation. Figure 5 presents the NDVI maps generated from Landsat data for 2001, 2011, and 2021, while vegetation cover was quantified using ERDAS Imagine 2015. In 2001, NDVI values within the study area ranged from −0.02 to 0.68; in 2013, from −0.06 to 0.66; and in 2021, from −0.06 to 0.66. Higher NDVI values correspond to agricultural land and mixed forest, indicating elevated vegetative productivity [67]. In contrast, lower NDVI values are associated with less productive surfaces such as bare soil, water bodies, and urban areas. Collectively, the NDVI patterns indicate a marked decline in productive vegetated cover across the study region.

### 4.4 Normalized Difference Built‐Up Index

**FIGURE 6:**
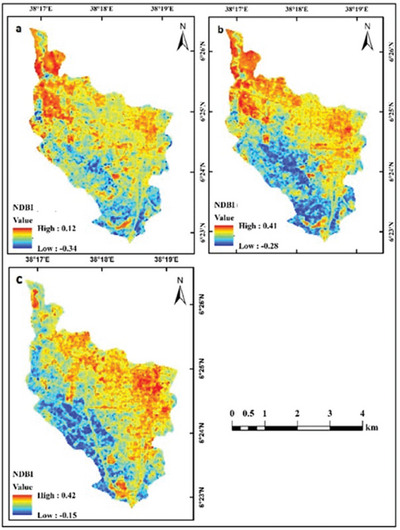
NDBI maps in (a) 2001, (b) 2011, and (c) 2021.

Figure 6 illustrates the spatial distribution of NDBI values for 2001, 2011, and 2021. Unlike low NDBI values characterize NDVI, densely vegetated and healthy vegetation zones. In 2001, NDBI values in the study area ranged from −0.34 to 0.12; in 2011 and 2021, they ranged from −0.28 to 0.41 and −0.15 to 0.42, respectively. Higher NDBI values correspond to urbanized and built‐up areas, while lower values correspond to non‐built‐up areas. The observed temporal trend shows a continuous increase in NDBI values from 2001 to 2021, indicating sustained expansion of built‐up land across the study area.

### 4.5 Land Surface Temperature Distribution

**FIGURE 7:**
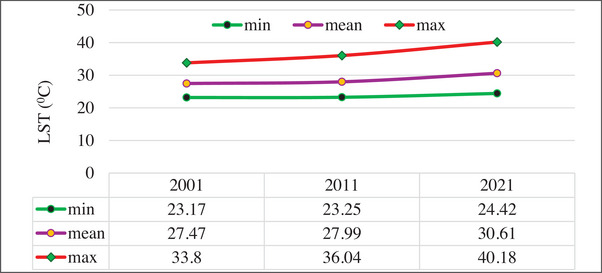
Minimum, Mean, and Maximum LST in 2001, 2011, and 2021.

Figure 7 shows the spatial configuration and areal distribution of LST in Dilla for 2001, 2011, and 2021. The observed patterns of LST and their temporal changes result from rapid LULC changes. In 2001, LST values ranged from 23.17°C to 33.8°C in 2011, from 23.25°C to 36.04°C; and in 2021, from 24.42°C to 40.18°C. Between 2001 and 2021, LST was found to increase in parallel with the growth of built‐up areas in the study area. The northern portion of the region has lower temperatures, reflecting the dominance of vegetation and forest cover. In contrast, the eastern sector is characterized by high LST, linked to rapid urban growth and the loss of vegetated land, indicating a positive relationship between LST and urbanization. The highest mean LST was 30.61°C in 2021, and the lowest was 27.47°C in 2001.

**FIGURE 8:**
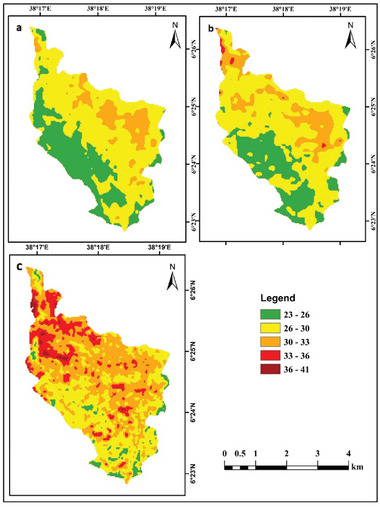
LST maps for the years a) 2001, b) 2011, and c) 2021.

The areal extent of the lowest LST class (23°C–26°C) declined markedly from 3.4281 km^2^ in 2001 to 0.5594 km^2^ in 2021, whereas the highest LST category (36°C–41°C) expanded from 0 km^2^ in 2001 to 14.2776 km^2^ in 2021. Detailed statistics for each LST interval over the period 2001–2021 are summarized in Table 6. Figure 8 shows the spatial distribution of the LST maps for the years a) 2001, b) 2011, and c) 2021.

**TABLE 6:** The area coverage of Land Surface Temperature.

LST Class	LST area coverage (km^2^)		
	2001	2011	2021
23–26	3.4281	1.8945	0.5594
26–30	8.3574	8.1558	5.1211
30–33	2.4921	4.1364	6.5485
33–36	0	0.09	1.8640
36–41	0	0	0.1846
Total	14.2776	14.2776	14.2776

### 4.6 Correlation Between Land Cover Index (LCI) and LST

**FIGURE 9:**
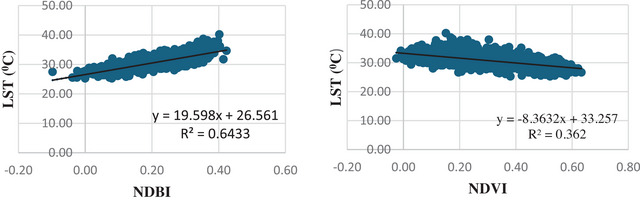
Correlation between LST and LCI for 2021.

Figure 9 shows the relationships between the LST and LCIs for LST prediction. In 2021, LST showed a significant positive relationship with NDBI (R^2^ = 0.643), which implies that built‐up intensity is a major driver of surface warming. By contrast, NDVI was negatively correlated with LST (R^2^ = 0.362), indicating cooler conditions in vegetated areas, but with less explanatory power than NDBI. Accordingly, NDVI is a less effective predictor of LST than NDBI in the study area.

### 4.7 Land Use Land Cover Simulation

**FIGURE 10:**
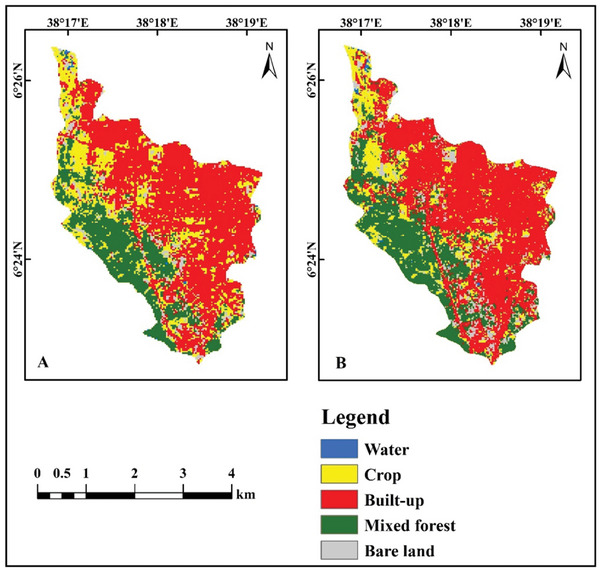
LULC map of a) classified and b) predicted for 2021.

A visual comparison showed good agreement between the LULC pattern classified by the SVM model and the 2021 LULC pattern simulated by the CA‐Markov model (Figure 10). Table 7 compares the areal extent of each LULC class between the classified and simulated 2021 maps, showing that corresponding classes occupy broadly similar areas. The observed and simulated built‐up classes covered 7.400 and 7.620 km^2^, respectively, indicating that the projected built‐up extent slightly overestimated the actual built‐up area in 2021. For water bodies, the area on the classified map was 0.057 km^2^, compared with 0.0555 km^2^ on the projected 2021 map.

**TABLE 7:** Area coverage of observed and predicted LULC of 2021.

Land use types	Total area (km^2^)	
	Observed	Predicted
Water	0.0576	0.055
Bare land	0.9315	1.256
Built‐up	7.400	7.620
Mixed forest	2.870	3.321
Crop	3.017	2.173
Total	14.277	14.277

The transition probability matrices for the years 2001–2011 and 2011–2021 are given in Table 8. The diagonal elements are the probability that each land‐use/land‐cover class will stay in its original land‐use/land‐cover class during the respective interval. As shown in Table 7, the built‐up class has the highest persistence with probabilities of 0.84 for 2001–2011 and 0.85 for 2011–2021. By contrast, water bodies have much lower chances of not changing, with probabilities of 0.09 and 0.11 for 2001–2011 and 2011–2021, respectively, which is greater instability than other classes. The off‐diagonal elements represent the probabilities of transition from one LULC class to another for the specified periods.

**TABLE 8:** Transition probability matrix for the prediction of 2021.

	Land use type	Water	Bare land	Built‐up	Mixed forest	Crop
2001–2011	Water	**0.0918**	0.0149	0.2060	0.3275	0.3598
	Bare land	0.0000	**0.4195**	0.3366	0.0024	0.2415
	Built‐up	0.0000	0.0310	**0.8424**	0.0025	0.1241
	Mixed forest	0.0026	0.0052	0.1130	**0.8069**	0.0722
	Crop	0.0038	0.0419	0.4735	0.1054	**0.3754**
2011–2021	Water	**0.1176**	0.0588	0.2157	0.2941	0.3137
	Bare land	0.0000	**0.2860**	0.3880	0.0400	0.2860
	Built‐up	0.0003	0.0307	**0.8597**	0.0268	0.0825
	Mixed forest	0.0041	0.0970	0.2884	**0.5261**	0.0843
	Crop	0.0078	0.0951	0.3718	0.1403	**0.3850**

The built‐up class exhibits the highest level of agreement between the simulated and classified 2021 LULC maps (KIA = 0.95) (Table 9), whereas the water‐body class shows the lowest correspondence (KIA = 0.72). These validation results provided sufficient confidence in the model to project future land‐use and land‐cover patterns for the year 2031.

**TABLE 9:** KIA of each LULC class.

LULC class	Water	Bare land	Built‐up	Mixed forest	Crop
KIA	0.72	0.87	0.95	0.85	0.85

### 4.8 Prediction of Future LULC for 2031

**FIGURE 11:**
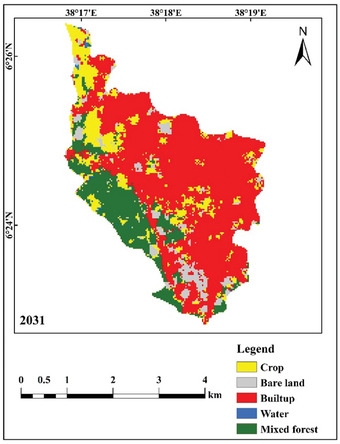
Predicted LULC map of the study area for the year 2031.

Figure 11 depicts the anticipated land use and land cover map of the study region for 2031. Over the coming decades, the developed area is projected to grow by 1.391 km^2^, while unutilized land is likely to increase by 0.173 km^2^. Conversely, the extent of water bodies, mixed forests, and agricultural land is anticipated to diminish by 0.012, 0.5139, and 0.905 km^2^, respectively. Table 10 and Figure 11 provide a comparative assessment of the areal extent of each LULC class for 2021 and the projected scenario for 2031.

**TABLE 10:** Predicted area coverage of 2031.

LULC area coverage in km^2^		
Land use types	2021	2031
Water	0.057	0.0450
Bare land	0.931	1.105
Built‐up	7.400	8.792
Mixed forest	2.870	2.356
Crop	3.017	2.112
Total	14.277	14.277

### 4.9 Prediction of Future LST for 2031

**FIGURE 12:**
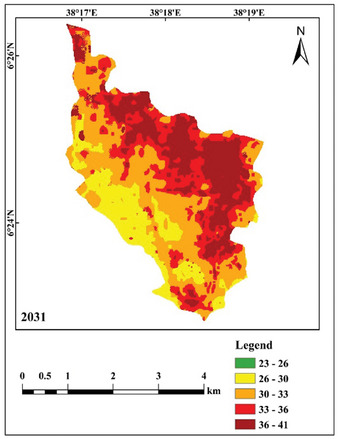
Predicted LST map of the study area for the year 2031.

Figure 12 depicts the anticipated LST map for the study region in 2031. The lowest land surface temperature group (23°C–26°C) is projected to diminish, whilst the highest land surface temperature category (36°C–41°C) is forecast to rise. The projected reductions in area for the initial three LST categories (23°C–26°C, 26°C–30°C, and 30°C–33°C) are 0.557, 4.342, and 5.690 km^2^, respectively. In contrast, the lower temperature ranges (33°C–36°C) and (36°C–41°C) are anticipated to expand by 9.069 and 1.519 km^2^, respectively, from 2021 to 2031. Table 11 presents a comparative analysis of the regions included in each LST category for 2021 and 2031.

**TABLE 11:** Area coverage of observed and predicted LST in 2021 and 2031.

LST class	LST area coverage (km^2^)	
	2021	2031
23–26	0.559	0.003
26–30	5.121	0.780
30–33	6.549	0.859
33–36	1.864	10.933
36–41	0.185	1.703
Total	14.278	14.278

## 5 Discussion

This study used a Cellular Automata—Markov chain approach to predict seasonal LULC and seasonal LST in Dilla Town, southern Ethiopia. Analysis of LULC dynamics in Dilla revealed a rising demand for land driven by rapid urban expansion. Given the complexity of urban LULC change, SVMs were used as a high‐accuracy classifier and applied to the years 2001, 2011, and 2021. Tamirat et al. [37] found that SVM‐based classifications are suitable for capturing complex rural‐urban landscape transitions and spatial patterns, and Kebede et al. [28] demonstrated that SVM can produce high‐precision LULC maps with accuracies exceeding 80% [28, 37]. Overall, the SVM algorithm has shown good performance in LULC classification compared with Parallelepiped, Minimum Distance, Maximum Likelihood (MLC), Artificial Neural Network (ANN), and Mahalanobis Distance classifiers [36, 46]. The built‐up area expanded from 2.058 km^2^ in 2001 to 7.401 km^2^ in 2021 and is projected to reach 8.792 km^2^ by 2031 [43]. This trend is consistent with findings from other parts of Ethiopia, including Bahir Dar [16]. Addis Ababa [1] and southwestern Ethiopia [23]. The accuracy assessment comparing the classified and modeled LULC maps for 2021 revealed high concordance, with a Kappa Index of Agreement of 0.95, confirming the reliability of the projected land‐use and land‐cover changes for 2031.

The predictive power of NDBI and NDVI for land‐cover‐based spatiotemporal patterns of temperature has been extensively tested [38, 68]. NDVI is used to characterize vegetation because healthy plants strongly reflect near‐infrared radiation and absorb red light. In contrast, NDBI is obtained from the SWIR and NIR bands to identify urban and built‐up areas. Correlation analysis between LST and the spectral indices NDVI, NDBI, and NDBaI indicates a significant negative association with NDVI and strong positive associations with both NDBI and NDBaI. In the present study, NDBI was identified as the primary predictor of the spatial distribution of LST (R^2^ = 0.64), and a linear regression model was employed to forecast urban expansion and the associated spatio‐temporal temperature patterns. Numerous studies also report a strong positive association between NDBI and LST [19, 24]. Our diagnostic analysis further confirmed that NDBI gives the most robust explanation of temperature variability among the tested indices.

The CA–Markov chain framework demonstrated robust capability to predict seasonal land use and land cover classes, aligning with recent time‐series‐based spatial pattern identification approaches. Markov chain–based modeling supports the prediction of environmental transitions and yields important insights into potential changes in surface thermal conditions driven by shifts in vegetation cover [47]. In this study, the approach was efficient for estimating temperature variability at the exact spatio‐temporal resolution as the LULC patterns, thereby allowing coherent modeling of regional processes such as urban surface dynamics.

The Markov chain model exhibits great potential for predicting future LST, and merits further application, as it is effective for tracking land‐use and land‐cover change, and is computationally efficient, flexible, and conceptually simple [53, 68, 69, 70]. The current study is critical because it gives a framework to predict future thermal conditions in Dilla Town based on historical urban growth trajectories [61, 71]. Numerous studies worldwide have demonstrated that urbanization significantly influences LST and is governed by nonlinear landscape‐driven mechanisms [72, 73]. Accordingly, forecasting the expansion of the urban area and its impact on the thermal environment of Dilla town using remote sensing data is crucial. This approach can inform local adaptation strategies, enhance temperature‐related planning and decision‐making, and support more balanced urban development that explicitly considers the microclimatic consequences of land‐use and land‐cover change.

The NDBI is used as a benchmark for urbanization and its anticipated expansion to forecast future variations in LST distribution. The findings suggest that the lower land surface temperature (LST) group (23°C–26°C) is anticipated to diminish, whilst the region included by the highest LST category (36°C–41°C) is forecasted to expand by 2031. The developed areas and barren terrain showed the highest LST, whereas aquatic environments, forests, and agricultural land showed the lowest LST. In the 2031 forecast, the developed region is expected to exhibit an elevated land surface temperature, especially in the eastern section of the study area. This discovery aligns with several studies on future land surface temperature modeling [61, 74]. The forecasted outcomes indicate that LST may increase in regions exhibiting consistent LULC from 2021 to 2031. The rise in land surface temperature trends is a consequence of land‐use and land‐cover changes, characterized by the expansion of urban areas at the expense of diminishing green spaces and wetlands.

### 5.1 Limitations of this Study

This study has the following limitations. First, LST was retrieved using a single‐channel algorithm with NDVI‐based emissivity (TIRS Band 10/TM thermal). This widely used but atmosphere‐sensitive configuration does not exploit split‐window correction and can propagate uncertainties associated with water vapor content and emissivity assignment. Second, the 30 m spatial resolution of the Landsat data aggregates heterogeneous urban materials within individual pixels, potentially biasing both spectral indices and LST estimates in fine‐grained urban fabrics. Third, the use of single‐month/epoch imagery captures long‐term (decadal) changes but does not resolve intra‐annual or seasonal thermal variability. Future work could improve LST retrievals by applying split‐window techniques (Bands 10–11) or radiative‐transfer‐based atmospheric corrections and by integrating multi‐sensor thermal datasets to better account for atmospheric variability and emissivity heterogeneity.

Broader temporal sampling—multi‐season and multi‐year stacks—would quantify seasonal urban heat island dynamics and extreme heat exposure beyond decadal snapshots. Higher spatial detail, achieved through fusion with finer‐resolution optical/thermal sources, could resolve micro‐morphologies that modulate neighborhood‐scale LST. In contrast, process‐rich or hybrid change models (agent‐based, ML ensembles) and expanded in‐situ validation would capture socio‐economic drivers, non‐linearities, and surface/air temperature contrasts more faithfully. Finally, planning‐oriented scenarios that test greening, wetland protection, and reflective‐surface interventions should be prioritized for the eastern growth corridors identified as thermal hotspots.

## 6 Conclusion

This study demonstrated that rapid urban expansion in Dilla Town between 2001 and 2021 has driven substantial LULC change, characterized by the conversion of mixed forest, cropland, and water bodies into built‐up and bare land, with a corresponding intensification of LST. The strong positive relationship between NDBI and LST, alongside the negative association between NDVI and LST, confirms that increasing impervious surface cover is the dominant driver of surface warming, while vegetation mitigates thermal stress. The CA–Markov chain model reproduced the 2021 LULC pattern with high agreement (KIA ≈ 0.95), lending confidence to projections for 2031 that indicate continued urban growth, further loss of vegetated and agricultural areas, and a marked shift toward hotter LST classes. These findings highlight the need to integrate heat‐aware LULC planning into municipal decision‐making by conserving remaining green and blue infrastructure, promoting compact, climate‐sensitive urban growth, and encouraging the use of high‐albedo, permeable materials in new developments. The established CA–Markov–LST framework should be used as a planning tool to test alternative development scenarios and their thermal implications before implementation. At the same time, ongoing monitoring with multi‐temporal remote sensing, complemented by ground‐based temperature measurements, is recommended to refine local heat‐risk assessments. Overall, the results underscore that proactive, evidence‐based urban planning and targeted greening interventions are essential to moderate future surface warming, enhance thermal comfort, and support climate‐resilient urban development in Dilla Town.

The detected link between LULC transitions (conversion of vegetated or agricultural surfaces to impervious built‐up land) and rising land surface temperature is governed by broadly applicable biophysical processes, including reduced evapotranspiration, modified surface albedo, and increased heat storage in urban materials. These mechanisms operate in many rapidly urbanizing landscapes, indicating that similar LST responses can be expected where comparable patterns of urban expansion occur. The analytical framework—multi‐temporal Landsat‐based LULC classification, LST retrieval from thermal bands, and CA–Markov modeling for scenario projection—is transferable to other regions with suitable remote‐sensing coverage. However, the magnitude and spatial configuration of predicted changes may vary with local controls such as climate regime, background vegetation, topography, urban morphology, and planning policies; therefore, regional calibration and validation are recommended when applying the approach elsewhere.

## Conflicts of Interest

The authors declare no conflicts of interest.

## Data Availability

The data that support the findings of this study are available from the corresponding author upon reasonable request.
